# Infliximab Exerts No Direct Hepatotoxic Effect on HepG2 Cells In Vitro

**DOI:** 10.1007/s10620-012-2159-7

**Published:** 2012-04-26

**Authors:** Hilbert S. de Vries, Tineke de Heij, Henie M. J. Roelofs, Rene H. M. te Morsche, Wilbert H. M. Peters, Dirk J. de Jong

**Affiliations:** Department of Gastroenterology and Hepatology, Radboud University Nijmegen Medical Centre, PO Box 9101, 6500 HB Nijmegen, The Netherlands

**Keywords:** Inflammatory bowel disease, Hepatotoxicity, HepG2 cells, Infliximab, Methotrexate, Azathioprine, 6-mercaptopurine, 6-thioguanine

## Abstract

**Background:**

Infliximab-induced hepatotoxicity is reported in several case studies involving patients with inflammatory bowel disease (IBD) and a direct hepatotoxic effect has been proposed.

**Objective:**

The aim of this study was to determine the direct in vitro toxicity of infliximab. As a proof of principle the in vitro toxicity of thiopurines and methotrexate was also determined.

**Methods:**

Cell survival curves and the half maximal inhibitory concentrations (IC_50_) were obtained after 24, 48 and 72 h of incubation in HepG2 cells with the IBD drugs azathioprine, 6-mercaptopurine, 6-thioguanine, methotrexate or infliximab by using the WST-1 cytotoxicity assay.

**Results:**

No in vitro hepatotoxicity in HepG2 cells was seen with infliximab, while concentration-dependent cytotoxicity was observed when HepG2 cells were incubated with increasing concentrations of azathioprine, 6-mercaptopurine and 6-thioguanine.

**Conclusion:**

Infliximab alone or given in combination with azathioprine showed no direct hepatotoxic effect in vitro*,* indicating that the postulated direct hepatotoxicity of infliximab is unlikely.

## Introduction

Hepatotoxicity is defined as injury to the liver that is associated with impaired liver function caused by exposure to a drug or another noninfectious agent [1]. It is a serious complication, frequently observed in the medical treatment of inflammatory bowel disease (IBD) [2]. It can be directly attributed to the type of drugs used to treat IBD, such as immunosuppressants or biological therapies targeting TNF-α. However, hepatotoxicity may also result from drugs used to treat complications of immunosuppressants and TNF-α antagonists, e.g. isoniazid for the treatment of reactivation tuberculosis, or may be a result of exacerbation of underlying chronic viral hepatitis caused by immunosuppression [3].

In December 2004, the Food and Drug Administration (FDA) issued a drug warning to alert health care professionals to the risk of hepatotoxicity linked to infliximab [4]. However, severe hepatic reactions, including acute liver failure, jaundice, hepatitis or cholestasis have rarely been reported in patients receiving infliximab [5–8]. Furthermore, as demonstrated by Sokolove et al. [6] elevations of serum transamines in patients receiving biological therapy are uncommon and abnormalities of more than two times the upper limit of normal are rarely observed. The mechanism of infliximab-induced hepatotoxicity is poorly understood, although a direct hepatoxic effect has been proposed by several authors [6–8]. To our knowledge, no in vivo or in vitro results supporting this hypothesis have been reported.

The aim of this study was to determine the in vitro hepatotoxicity of infliximab. As a proof of principle, the conventional IBD medication, i.e. the thiopurines azathioprine, 6-mercaptopurine and 6-thioguanine and methotrexate, which are all known to be hepatotoxic, were also tested. Although cultures of primary human hepatocytes seem to have the most relevant physiological properties for the evaluation of in vitro IBD drug hepatotoxicity, they are difficult to obtain and rapidly lose their metabolic properties [9]. Therefore, we used a human liver hepatocellular carcinoma (HepG2) cell line, which is very stable, easy to handle and previously used in drug toxicity studies [9]. HepG2 cells were incubated with increasing concentrations of infliximab, methotrexate or thiopurines for 24, 48 or 72 h and subsequently cell viability was determined.

## Materials and Methods

### Cell Culture

Human hepatocellular carcinoma (HepG2) cells (American Type Culture Collection, Rockville, Maryland, USA) were grown in Dulbecco’s Modified Eagle Medium (DMEM, PAA Laboratories GmbH, Pasching, Austria) containing 10 % (v/v) heat-inactivated fetal bovine serum (Gibco Invitrogen, Paisley, Scotland), 1X non-essential amino acids (PAA), 20 mM HEPES buffer (PAA) and 50 mg/l gentamycin (Gibco) at 37 °C in a humidified atmosphere containing 5 % CO_2_. The medium was renewed every 3 days and when confluence was reached, cells were harvested with trypsin/EDTA (Cambrex, Verviers, Belgium), washed with phosphate-buffered saline, and used for cytotoxicity assays.

### Cytotoxicity Assays

HepG2 cells were seeded in flat-bottomed 96-well microtitre plates (Costar; Corning Inc., Corning, New York, USA) at a density of 5.0 × 10^4^ cells per well in a final volume of 100 μl culture medium, and cells were cultured for 24 h. Subsequently, cells were incubated with the single drugs azathioprine, 6-mercaptopurine, methotrexate (all Sigma-Aldrich Chemie B.V., Zwijndrecht, The Netherlands), 6-thioguanine (Alfa Aesar GmbH & Co KG, Karlsruhe, Germany) or infliximab (Remicade^©^, Centocor, Leiden, the Netherlands) for 24, 48 and 72 h. The following concentration series were used: azathioprine: 0.002 μM–4 mM; 6-mercaptopurine: 0.002–200 μM; 6-thioguanine: 0.002 μM–4 mM; methotrexate: 50 nM–100 μM; infliximab: 0.002 mg/l–5 g/l.

In subsequent experiments the cytotoxicity of a combination of drugs was tested. A single, non-toxic concentration of azathioprine (1 μM) was tested in combination with a concentration range of infliximab (0.002 mg/l–5 g/l), whereas a single, non-toxic concentration of infliximab (312 mg/l) was tested in combination with a concentration range of azathioprine (0.002 μM–4 mM).

All drugs were first dissolved in 0.1 M NaOH and then rapidly diluted in the culture medium to reach the final concentration. A refreshment of the culture medium with the various concentrations of drugs was done every 24 h. After the incubation with drugs, a cell survival assessment was performed by adding water-soluble tetrazolium salt-1 (WST-1, Roche Diagnostics Nederland BV, Almere, The Netherlands) according to the manufacturer’s instructions. In the WST-1 assay, tetrazolium salts are cleaved by dehydrogenases of viable cells to produce formazan. The amount of formazan dye was quantified by using an ELISA plate reader at 440 nm. Cell survival was defined as:

$$ \text{Cell survival}=\frac{{A_{\text{experimental}} - A_{\text{background}}
}}{{A_{\text{control}} - A_{\text{background}} }} \times 100\,\%
$$

where *A*_experimental_ is the absorbance of drug incubated cells plus WST-1, *A*_background_ is the absorbance of culture medium plus WST-1 in the absence of cells and *A*_control_ is the absorbance of cells without drugs plus WST-1. Test results were obtained from three independent experiments, each performed in triplicate.

### Statistics

One-way ANOVA analysis was used to compare the effect of incubation time. A *p*-value <0.05 was considered statistically significant. Data were normalized to untreated cells using GraphPad Prism to calculate the concentration at which cell survival is 50 % (IC_50_, i.e. half maximal inhibitory concentrations). All statistical analyses and calculations were carried out using GraphPad Prism (version 4.0; GraphPad Software, San Diego, California, USA).

## Results

**Fig. 1:**
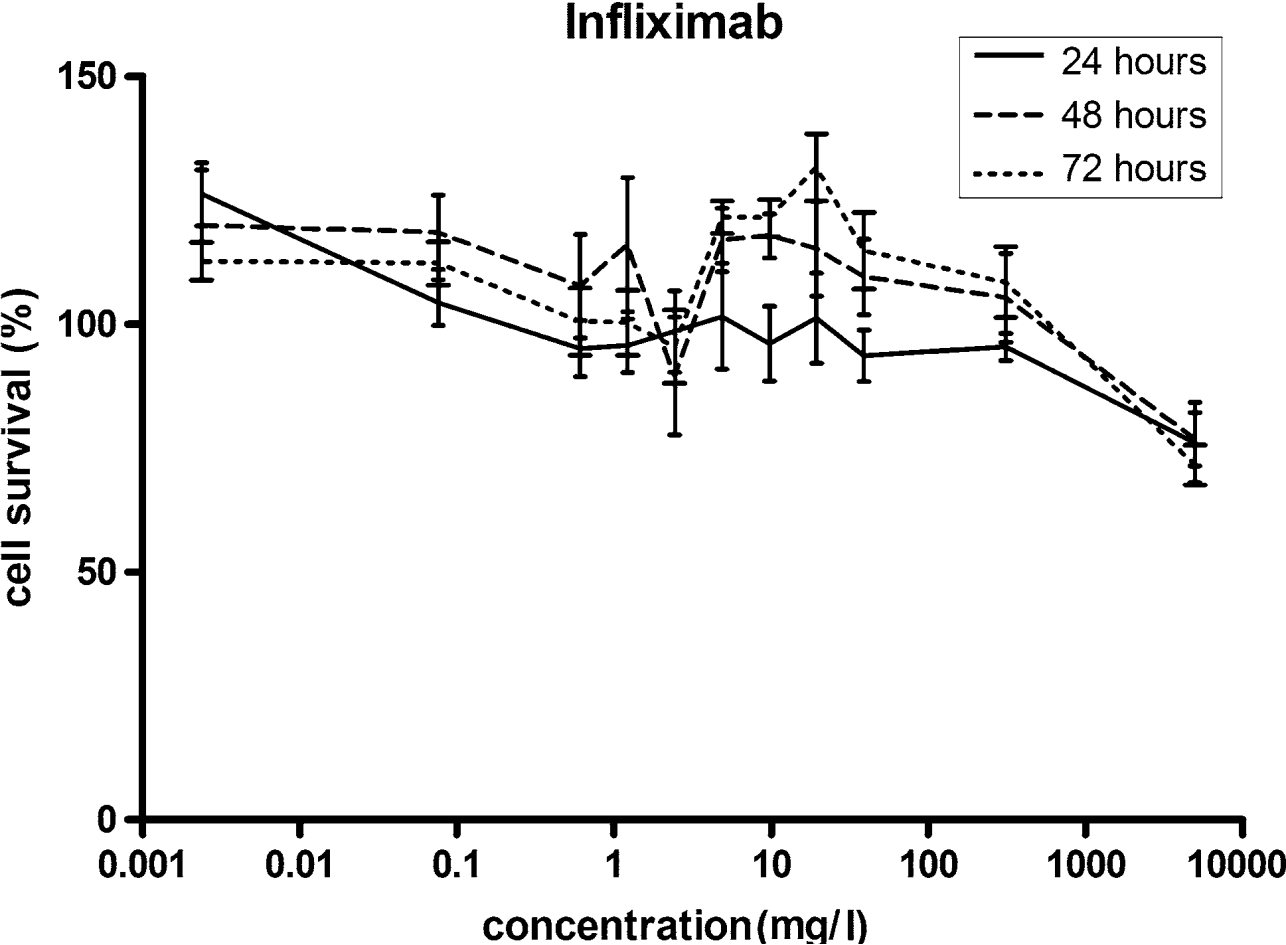
Cell survival of HepG2 cells after exposure to various concentrations of infliximab for 24, 48 or 72 h. Cell survival was expressed as a percentage of untreated cells. Values are means ± SEM of three independent experiments performed in triplicate

**Fig. 2:**
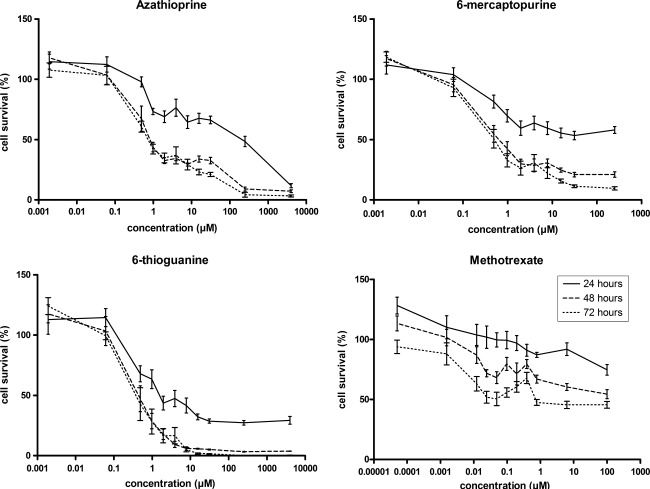
Effect of thiopurines and methotrexate on HepG2 cell viability. HepG2 cells were incubated with various concentrations of IBD drugs for 24, 48 or 72 h and cell survival was measured and expressed as percentage of untreated cells. The graphs summarize the results of three independent experiments (means ± SEM), performed in triplicate

The incubation of HepG2 cells with increasing concentrations of infliximab did not result in significant changes in cell viability, indicating that a direct in vitro cytotoxic effect was absent (Fig. 1). At all concentrations of infliximab tested after 24, 48 or 72 h of incubation, cell survival was above 50 % and no IC_50_ values could be determined (Table 1). Only at the highest concentration of 5 g/l, could a decrease of about 30 % in cell survival could be seen (Fig. 1). Concentration-dependent cytotoxicity was clearly observed when HepG2 cells were incubated with increasing concentrations of azathioprine, 6-mercaptopurine or 6-thioguanine (Fig. 2). Only methotrexate showed a time-dependent cytotoxic effect on HepG2 cells. Incubation with various concentrations of methotrexate for 72 h resulted in a significant difference when compared to 24 h of incubation (*p* < 0.01).

**Table 1:** Cytotoxicity of IBD drugs in HepG2 cells

Incubation	IC_50_ (μM)				
	Azathioprine	6-Mercaptopurine	6-Thioguanine	Methotrexate	Infliximab
24 h	33.7 ± 29.3	3.8 ± 4.1	1.1 ± 0.8	NA	NA
48 h	0.5 ± 0.3	0.5 ± 0.6	0.4 ± 0.5	9.3 ± 16.0	NA
72 h	0.9 ± 0.6	0.4 ± 0.3	0.5 ± 0.7	0.04 ± 0.03	NA

*IBD* inflammatory bowel disease, *NA* not applicable (IC_50_ value was not reached), *SD* standard deviation,* IC*_*50*_ half maximal inhibitory concentrations

Values are mean IC_50_ ± SD (*n* = 9) derived after normalization of dose response curves using Graphpad Prism

After 24 h of incubation, neither the combination of infliximab (0.002 mg/l–5 g/l) with a low dose (1 μM) of azathioprine nor the combination of a single, non-toxic concentration of infliximab (312 mg/l) in combination with azathioprine (0.002 μM–4 mM) showed any difference in cell survival (data not shown).

## Discussion

In this study infliximab showed no direct cytotoxic effect on HepG2 cells, even at concentrations far exceeding the maximum concentration of 118 μg/ml, which infliximab achieves when administered intravenously at a dosage of 5 mg/kg [10]. Concomitant incubation with both infliximab in different dosages and azathioprine at a non toxic concentration did not alter HepG2 cell viability. Our in vitro results therefore suggest that a direct hepatotoxicity of infliximab is implausible. Alternatively, infliximab-induced hepatotoxicity is more likely to be immuno-mediated or induced via Fc receptor-mediated interactions. After forming an immune complex with TNF-α, this complex is cleared by the mononuclear phagocytic system in the liver via Fc receptor-mediated interactions that in turn can activate Kupffer cells. These resident macrophages of the liver located in hepatic sinusoids do release reactive oxygen species which may lead to local damage of hepatocytes [11–13]. During infliximab therapy, increased formation of anti-nuclear antibodies has been observed [14], most possibly due to the fact that binding of infliximab to transmembrane TNF on the cell surface induces apoptosis, leading to the release of nucleosomes and the generation of anti-nuclear antibodies [15]. Since antibodies to TNF-α delay the repair of liver injury [16, 17], the use of infliximab might also exacerbate a previous suboptimal liver condition not recognized by any clinical symptoms or biochemical markers. Furthermore, a potential hepato-protective effect of TNF-α induced by increasing hepatocyte regeneration and decreasing apoptosis has been observed in a transgenic mouse model of chronic hepatitis C while treatment with anti-TNF-α blocked the anti-apoptotic and regenerative effects induced by TNF-α [18].

In contrast to our experience with infliximab, we observed a concentration dependent cytotoxic effect of the thiopurines in HepG2 cells, while methotrexate demonstrated a time- and concentration-dependent effect. The in vitro hepatotoxic effects of thiopurines have also been demonstrated by Petit et al*.*, comparing the cytotoxicity of thiopurines in human hepatocytes and HepaRG cells, incubated for 24, 48, 72 and 96 h with 1, 5 or 25 μM of azathioprine, 6-mercaptopurine or 6-thioguanine. They reported a dose- and time-dependent cytotoxic effect of azathioprine and 6-mercaptopurine in both human hepatocytes and HepRG cells, while 6-thioguanine had no significant effect on human hepatocytes. However, 72 h of incubation with either 5 or 25 μM of 6-thioguanine showed a 30 % decrease in cell survival of HepaRG cells [19].

The observed time-dependent cytotoxic effect of methotrexate in our study is in line with results of Yin et al. [20] who reported a time- and concentration-dependent effect of high dose methotrexate (1–10 mM) in rat hepatocytes. These concentrations however go far beyond the mean peak concentration in human plasma of 1.14 μM achieved after subcutaneous administration of 15 mg methotrexate to patients with IBD [21].

Several limitations of our study should be noticed. First of all, results of in vitro studies cannot be directly extrapolated to the in vivo situation. Isolated liver (carcinoma) cells will respond differently to stress or toxic compounds than to an intact and perfused liver. Therefore, although results from cell lines add to the understanding of drug-induced toxicity, they will be difficult to translate into clinical practice. Processes of absorption, distribution, metabolism and excretion, which determine the exposure of the target tissues of an organism in vivo, are mainly absent in in vitro studies [22]. Furthermore, peripheral serum or plasma concentrations do not reflect the concentrations in the portal vein. Therefore the drug concentrations to which the liver is actually exposed are largely unknown. These points are especially true for the thiopurines. Resorption is highly variable in animal studies as well as in patients with inflammatory bowel disease and the pro-drugs azathioprine and 6-mercaptopurine are rapidly converted [23–26]. Additionally, as in hepatocytes in the primary culture, changes in the expression of drug metabolizing enzymes over time occur in HepG2 cells [27]. Therefore neither primary cultures of hepatocytes nor HepG2 cells display an ideal model mimicking the expression levels of drug metabolizing enzymes as present in hepatocytes in vivo, thereby limiting the reproducibility of in vitro hepatotoxicity experiments using different cell cultures [27]. In our study we focused on cell viability as a marker of hepatotoxicity. Alterations in pathways underlying cell death including oxidative stress were not studied.

In conclusion, our study suggests that infliximab does not have a direct toxic effect on HepG2 cells. In addition, infliximab in combination with thiopurines does not increase their in vitro toxicity on HepG2 cells. Our results may not be translated to clinical practice directly without considering the limitations of these findings. On the other hand, no alarming cytotoxicity is seen in the same assay that shows evident dose-related thiopurine cytotoxicity. Future studies regarding the hepatotoxic effects of infliximab should focus on Fc receptor-mediated interactions and auto-immune related factors.

## Abbreviations

IBD: Inflammatory bowel disease
CD: Crohn’s disease
UC: Ulcerative colitis
TNF-α: Tumour necrosis factor alpha
